# The Perfect Cup? Coffee-Derived Polyphenols and Their Roles in Mitigating Factors Affecting Type 2 Diabetes Pathogenesis

**DOI:** 10.3390/molecules29040751

**Published:** 2024-02-06

**Authors:** Brooke Chapple, Seth Woodfin, William Moore

**Affiliations:** Department of Biology and Chemistry, School of Health Sciences, Liberty University, Lynchburg, VA 24515, USA; bechapple@liberty.edu (B.C.); sfwoodfin@liberty.edu (S.W.)

**Keywords:** insulin resistance, β-cell function, enterodiol, enterolactone, matairesinol, secoisolariciresinol, kaempferol, quercetin, chlorogenic acid

## Abstract

Type 2 diabetes (T2D) is a growing health concern with an estimated 462 million people having been diagnosed worldwide. T2D is characterized by chronically elevated blood glucose and insulin resistance, which culminate in a diminished function of the β-cell mass in its later stages. This can be perpetuated by and result in inflammation, excess reactive oxygen species production, obesity, and the dysregulation of multiple cellular pathways. Many naturally occurring small molecules have been investigated in terms of their roles in modulating glucose homeostasis and β-cell function. Many of these compounds can be found in commonly used sources of food and drink. Interestingly, a correlation has been observed between coffee consumption and T2D incidence. However, the specific compounds responsible for this correlation and their mechanisms are still somewhat undetermined. This paper reviews recent research findings on the effects of several polyphenols that are either found in coffee or are metabolites of compounds found in coffee (enterodiol, enterolactone, matairesinol, secoisolariciresinol, kaempferol, quercetin, and chlorogenic acid) on glucose homeostasis and health complications associated with glucose dysregulation, with a special emphasis on their potential anti-diabetic effects. The factors that affect polyphenol content in coffee are also addressed.

## 1. Introduction

Diabetes affects roughly 11.6% of the U.S. population [1], with approximately 90% of these suffering from type 2 diabetes (T2D) [2]. T2D results from insulin resistance, which is directly linked to obesity [3]. Prior to the onset of T2D, insulin resistance leads to hyperinsulinemia as the pancreatic β-cells work to compensate for diminished insulin sensitivity. This ultimately culminates in an inability of the β-cells to produce sufficient insulin to overcome the lowered sensitivity, thus resulting in fasting hyperglycemia, which is clinically categorized as T2D [4]. The dysregulation of blood glucose levels is detrimental to a person’s overall health, as a chronically elevated blood glucose can lead to an array of secondary complications, including a state of chronic low-grade inflammation [5], chronic kidney disease [6], cardiovascular disease [7], retinopathy [8], and neuropathy [9], which often lead to a plethora of podiatric issues that can ultimately necessitate limb amputation [10]. The current treatments for T2D include diet modification, exercise, and pharmaceutical agents that work via several mechanisms, including promoting insulin sensitivity and production, promoting GLP-1 activity and longevity, and inhibiting hepatic gluconeogenesis and glucose reabsorption in the kidneys [11,12]. Despite the many advancements that have been made in our understanding of T2D pathogenesis and therapeutic approaches, it is expected that the incidence of this disease will increase by nearly 17% by 2030 [13]. Therefore, the search for novel low-cost treatments is still warranted.

Coffee consumption and T2D have been shown to be negatively correlated [14], which supports the rationale that the coffee bean, which is rich in polyphenols and other small compounds, might be a reservoir for such antidiabetic agents. Indeed, several polyphenols, which are either found in coffee or are metabolites of polyphenols found in coffee, have been shown to exhibit anti-diabetic effects. Coffee contains approximately 355 mg of polyphenols per 180 mL serving on average, which is nearly double that of green tea, making coffee the beverage with the highest total polyphenol content [15]. Polyphenols’ positive health effects have been extensively researched, but available research aiming to identify the causative link(s) of the negative correlation between coffee consumption and T2D risk is limited. It is unlikely that the correlation is due to caffeine, as studies have shown that decaffeinated coffee has nearly the same association with T2D risk as caffeinated coffee [16,17,18]. This article will outline the factors involved in T2D pathogenesis and its sequelae relevant to the effects of the discussed coffee-derived polyphenols. A review will then summarize the available research on the select polyphenols found in coffee that have been shown to elicit effects that pertain to these factors. The following compounds will be reviewed: enterodiol (EDL), enterolactone (ENL), matairesinol (MA), secoisolariciresinol (SL), kaempferol (KL), quercetin (QN), and chlorogenic acid (CA).

## 2. Molecular and Cellular Basis of T2D Pathogenesis

### 2.1. PI3K/Akt/GLUT4

**Figure 1:**
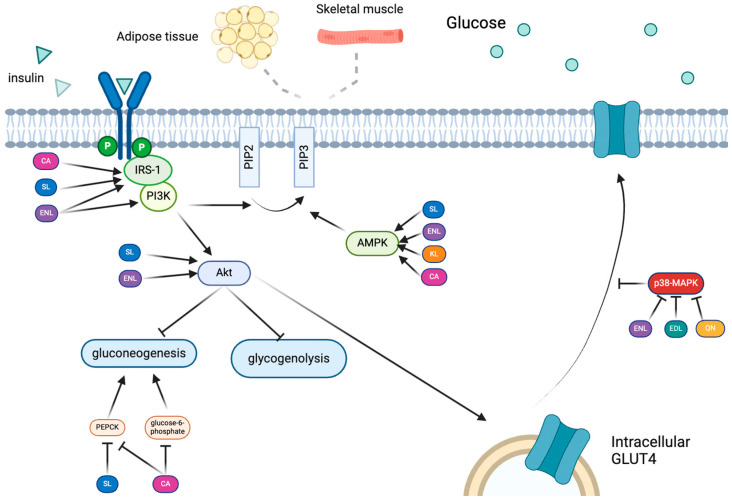
Suggested mechanisms by which the reviewed compounds regulate blood glucose levels in adipose, skeletal muscle, and/or liver tissue.

The phosphatidylinositol-3-kinase (PI3K) pathway is of major importance regarding glucose uptake and the insulin-stimulated translocation of glucose transporters, namely GLUT4, to the cell membrane [19]. The binding of insulin to its receptor leads to the recruitment and phosphorylation of insulin receptor substrates-1 and -2 (IRS-1 and -2), as the insulin receptor (IR) has a tyrosine kinase domain [20,21] (Figure 1). IRS phosphorylation allows for the recruitment of downstream effectors [22], including the p85 regulatory subunit of PI3K [23], which subsequently promotes the phosphorylation and activation of the serine/threonine kinase, Akt [19,24]. Akt phosphorylation has several downstream effects, which partly depend on the cell type. In the adipocytes and skeletal muscle, Akt phosphorylation leads to the translocation of glucose transporter 4 (GLUT4), a 12-transmembrane protein, to the cell membrane, resulting in the uptake of glucose into the cell from the bloodstream, thus lowering blood glucose levels [25]. T2D is characterized by the desensitization of the IR to insulin; thus, the tyrosine kinase activity of the IR is diminished [26]. As a result, none of the proteins mentioned in the above cascade effect are effectual to GLUT4 translocation and resultant glucose uptake. In the liver, Akt phosphorylation leads to the inhibition of glycogen synthesis and gluconeogenesis. Failure to respond to insulin thus may exacerbate hyperglycemia as glycogenolysis, and hepatic gluconeogenesis may persist even as the blood glucose levels are already elevated. 

In the β-cell, the activation of the PI3K/Akt pathway leads to insulin secretion, and a failure to activate PI3K/Akt signaling in the β-cell blocks insulin secretion [27,28]. Additionally, Akt and Bcl-2 are major players in the anti-apoptotic process, as Akt stimulates cell proliferation and Bcl-2 prevents cytochrome c release via mitochondrial pores, thus preventing the initiation of the caspase cascade resulting in apoptosis [29]. The activation of these proteins can be beneficial for the protection of islet β-cells, which are commonly damaged in people with type 2 diabetes [30].

### 2.2. GLUT2/cAMP/PKA/CREB/PDX-1

Glucose transporter 2 (GLUT2) is a relatively high-Km glucose transporter that is especially prevalent in hepatic and pancreatic tissues due to their roles in maintaining blood glucose homeostasis through gluconeogenesis, glycogenolysis, and insulin production and secretion. As it pertains to insulin secretion, when the blood glucose levels are elevated, GLUT2 facilitates glucose uptake into the pancreatic β-cells and activates a high-Km hexokinase (IV) or glucokinase. As the intracellular glucose levels rise, it is metabolized to the end of the generation of ATP. When the ATP:ADP ratio rises to a certain point, ATP binds to the Kir6.2 subunit of the ATP-sensitive potassium channel [31], thus blocking potassium entry into the cell. This leads to membrane depolarization and a subsequent influx of Ca^2+^. Elevated intracellular Ca^2+^ promotes cAMP formation by activating adenylyl cyclase. Increased cAMP activates protein kinase A (PKA) and cAMP-responsive element-binding protein (CREB) [32], the activity of which culminates in insulin secretion [33,34]. Either directly or indirectly, PKA activates pancreas duodenum homeobox-1 (PDX-1), which is an *slc2a2* (GLUT2) transcription factor [33,34,35]. It has been shown that the expression of PDX-1 restores the presence of functioning β-cells [36]. 

### 2.3. Inflammation and ROS

**Figure 2:**
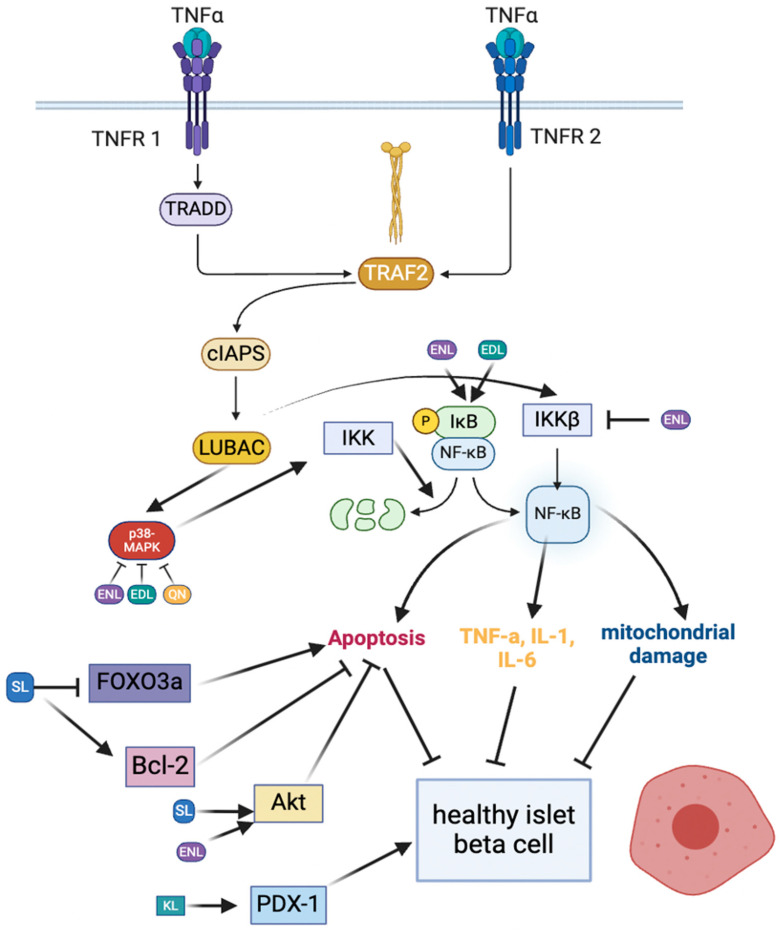
Select mechanisms by which the reviewed compounds decrease levels of pro-inflammatory cytokines, such as TNF-α, IL-1, or IL-6; minimize NF-κB expression; and promote healthy β-cell growth are indicated above.

Chronic low-grade inflammation is commonly associated with T2D and is thought to cause many of the clinical manifestations associated with the secondary complications of T2D [37]. Many of these complications are associated with increased oxidative stress and the apoptosis of islet β-cells [38]. The hypothalamus is responsible for the regulation of metabolic homeostasis largely through nutrient and appetite sensing via leptin and insulin sensors [39,40]. Studies have shown that a high-fat diet, as well as high glucose or lipid levels, can cause hypothalamic inflammation, which is thought to be a consequence of the hyperactivity involved in regulating the utilization of excess energy substrates [40]. The onset of detectable inflammation can occur within 24 h of a shift in diet [39]. This results in both insulin and leptin resistance [39,40], which leads to dysregulated satiety, energy use, and glucose homeostasis [40,41]. Chronic inflammation of the brain also leads to an overactivation of the sympathetic nervous system, which is largely associated with increased hepatic gluconeogenesis, further contributing to hyperglycemia and insulin release [40,42]. Hyperinsulinemia has been shown to further contribute to the progression of T2D and inflammation [40]. This pro-inflammatory status in people with type 2 diabetes can also contribute to an increased bodyweight and an imbalance in metabolic flexibility [40]. Two key mediators of these inflammatory effects are IκB kinase β (IKKβ) and tumor necrosis factor-α (TNF-α), which are pro-inflammatory cytokines that have been shown to actively contribute to brain inflammation [43]. This is thought to occur by the binding of TNF-α to its receptors, TNF receptor 1 (TNFR1; p55, and p60) and TNF receptor 2 (p75 and p80) [43,44] (Figure 2). When TNF-α binds to TNFR1, both receptor-interacting serine/threonine protein kinase 1 and TNFR1-associated death domain protein bind to the receptor on the cytosolic side of the membrane [43]. TNFR-associated factor 2, the cellular inhibitor of apoptosis protein 1 (cIAP1), cIAP2, and linear ubiquitin chain assembly complex (LUBAC) are brought together and produce ubiquitin chains that eventually cause the activation of MAPK and NF-κB [43]. These all work in harmony with each other, as the cIAP1 and cIAP2 proteins are bound by TGFβ-activated kinase 1-binding protein 2 (TAB2) and TAB3, which activate TGFβ-activated kinase 1 (TAK1), stimulating MAPK signaling [43]. The TAK1 protein is also necessary for the activation of IKKα and IKKβ on the ubiquitin chains produced by LUBAC, which, in turn, stimulates the NF-κB pathway [43]. The MAPK and NF-κB pathways lead to the increased expression of TNF-α and IL-1, 6, and 8, all of which are pro-inflammatory cytokines [45,46]. Thus, this is a positive feedback mechanism, which exacerbates systemic inflammation. The over-production of pro-inflammatory cytokines via the activation of the IKKβ (and thus, NF-κB) and MAPK pathways as well as the stimulation of apoptosis follows the activation of TNF-α receptors [43]. Pro-inflammatory mediators of TNF-α and IKKβ have been suggested as potential therapeutic targets for minimizing the inflammation of the hypothalamus [39,40].

It has also been well documented that reactive oxygen species (ROS) are avid contributors to the upregulation of proinflammatory cytokines [47]. The mechanisms by which ROS affects the hypothalamus have been extensively reviewed [39,40,47]. ROS are prime mediators of oxidative stress, especially in patients with diabetes, as they tend to be produced in excess [48]. This is due to the multifaceted nature of T2D pathogenesis, which includes the overactivation of NADPH oxidase and other related pathways stimulated by hyperglycemia [48,49]. This is a result of the nature of the electron transport system (ETS). Briefly, as the ETS produces ATP, electrons come to rest on O_2_, which results in the formation of H_2_O. A natural consequence of this is that a small percentage of O_2_ is partially reduced to a radical and converted to H_2_O_2_ by superoxide dismutase, which is also considered an ROS and toxic. Depending on the concentration of H_2_O_2_, the glutathione system, catalase, and thioredoxin/peroxiredoxin systems are typically sufficient to neutralize H_2_O_2_. However, excess H_2_O_2_ can be caused by chronic positive energy balance and a lack of physical activity, as is consistent with the typical lifestyle of a person with type 2 diabetes. Indeed, H_2_O_2_ and other ROS were found in excess in diabetic mouse models in a recent study [48]. This led to an increased production of pro-inflammatory cytokines, including IL-6 and TNF-α, because of the excessive movement of NF-κB to the nucleus [48]. It is hypothesized that this occurs through the activation of serine kinase IB kinase (IKK) by ROS/TNF-α [50]. IKK then degrades IκB, activating NF-κB and allowing it to continue to stimulate the production of pro-inflammatory mediators [51]. The overstimulation of ROS production, including H_2_O_2_, can cause mitochondrial damage by promoting the oxidation of mtDNA, which has been shown by subsequent increases in 8-hydroxydeoxyguanosine levels [52]. This ultimately leads to mitochondrial damage and an increased rate of apoptosis [52]. The mechanisms by which this occurs have been well reviewed [52,53,54]. It is partially due to the increased activity of NADPH oxidase due to a protein kinase C-dependent mechanism stimulated by hyperglycemia [55]. It is also due to the nature of the ETS, as described above. One of the pathways that is most adversely affected by mitochondrial damage, especially in conjunction with a pro-inflammatory status, is the PI3K/Akt signaling pathway [19]. Additionally, ROS damages the cell membranes through the peroxidation of unsaturated fatty acids, allowing for the passing of proteins that would otherwise be impermeable [56].

### 2.4. AMPK

Adenosine monophosphate-activated protein kinase (AMPK) regulates glucose uptake and the oxidation of lipids by coordinating the response to hormones such as leptin and adiponectin [57,58]. Leptin and adiponectin are adipokines, which, when elevated, have been shown to increase and decrease T2D risk, respectively [35]. Adiponectin activates AMPK, stimulating glucose uptake [59] as AMPK triggers the transition from PIP2 to PIP3, thus activating PI3K and initiating the GLUT4 translocation pathway, ultimately leading to glucose uptake [60] (Figure 1). In addition, high levels of adiponectin are largely related to decreased plasma triacylglycerol levels, which are typically high in patients with type 2 diabetes and contribute to obesity [61]. The effect of adiponectin is inhibited by TNF-α [59]. Therefore, its regulation by the AMPK pathway is critical for metabolic regulation and glucose uptake. 

High leptin levels, on the other hand, correlate with the progression of T2D [62] as it is associated with the regulation of energy expenditure and satiety signaling [62]. Excess leptin signaling normally lowers appetite; however, in T2D, leptin resistance can cause satiety confusion [62]. The failure to suppress appetite in the presence of high glucose levels typically leads to over-eating, which leads to obesity [62]. Additionally, the binding of leptin to its receptor indirectly causes p70S6K to phosphorylate Ser491 on the alpha subunit of AMPK, inhibiting its effects in the hypothalamus [63]. Not surprisingly, both adiponectin and leptin are related to hypertension and blood pressure regulation as well [64]. Thus, the poor regulation of these hormones and of the AMPK pathway contribute to hyperglycemia, obesity, and hypertension, all of which are common complications associated with T2D. 

### 2.5. MAPK Pathway (JNK Family)

Some studies show that the inhibition of the p38 mitogen-activated protein kinase (MAPK) has the potential to stimulate glucose uptake in adipocytes by increasing GLUT4 translocation and delaying insulin resistance in myotubes under high oxidative stress [65] (Figure 1). The p38 MAPK subunit is necessary for the phosphorylation of p300, an acetyltransferase [66]. This allows a complex to form between p65 and p300, which then allows for the acetylation of p-65-K310 [66]. This acetylation step is critical for the activation of NF-κB, which promotes inflammation through the production of TNF-α, IL-1, and IL-6 [46]. The p38 MAPK pathway is typically activated when cells are stressed, sometimes due to excess ROS production and inflammation, both of which are typical of T2D pathogenesis [67].

### 2.6. Pancreatic β-Cell Function

The primary role of pancreatic β-cells is the production and secretion of insulin into the bloodstream [68]. Because chronic hyperglycemia in people with type 2 diabetes increases the demand for insulin, the β-cells become increasingly overworked [69]. The activation of MTORC1 is largely responsible for this increased β-cell function and mass [70]. Eventually, the β-cells are no longer able to keep up with the body’s demand for insulin, leading to β-cell dysfunction, impaired autophagy, oxidative stress, and mitochondrial damage [69,70].

T2D causes a decline in functional β-cell mass [30], as chronically elevated blood glucose levels promote the secretion of IL-1β [71] and NF-κB-activated apoptosis [72]. In addition, the exposure of pancreatic β-cells to excessive ROS may cause DNA damage [73]. The hydroxyl radicals remove hydrogen atoms from the deoxyribose sugar or oxidize the DNA bases, which can cause DNA strand breakage and fragmentation [74]. This damage can lead to either cell cycle arrest or, in more extreme cases, apoptosis [75]. It has also been shown that excessive extracellular saturated fatty acid accumulation promotes β-cell apoptosis through the activation of the p38 MAPK pathway [76,77] (Figure 2). This is a common phenomenon among people with type 2 diabetes due to the decreased activity of lipoprotein lipase (LPL), whose major role is to break down blood triacylglycerols [78,79]. This occurs in T2D because LPL is primarily regulated by insulin, to which the body has become increasingly resistant [78]. The decline in islet β-cells, whether it is directly due to their overuse, the stimulation of certain pathways by hyperinsulinemia or fatty acid accumulation, or the presence of ROS, results in the dysregulation of blood glucose levels. This is largely due to insulin resistance and the subsequent inability to produce enough insulin to compensate for the resistance. 

### 2.7. Obesity

The majority of insulin-stimulated glucose uptake takes place in skeletal muscle [80]. However, chronic overnutrition and a positive energy balance, which is consistent with the lifestyle of most people with type 2 diabetes, promotes increased glucose disposal in adipocytes in which, after meeting the energy demand of the adipocyte, will ultimately be converted to and stored as fat [81]. 

This results in excess triacylglycerols and, eventually, obesity [82], which is linked to the excess production of pro-inflammatory cytokines and the activation of apoptotic pathways [83]. For example, the NF-κB pathway is initiated, yielding the production of many pro-inflammatory cytokines such as TNF-α, IL-1, and IL-6, thus perpetuating the problems of T2D [46,83,84] (Figure 2).

### 2.8. Hepatic Gluconeogenesis

Hepatic gluconeogenesis is the synthesis of glucose from non-carbohydrate sources and largely takes place in the liver, typically during periods of prolonged fasting, to prevent hypoglycemia [85]. During prolonged fasting, this becomes the main source of glucose, which allows for the preservation of muscle protein and the supplying of glucose to obligatory glucose users [4] following the depletion of hepatic glycogen stores. Gluconeogenesis is regulated by four enzymes that allow for the bypassing of the three non-equilibrium reactions in glycolysis. Thus, gluconeogenesis is essentially reversed glycolysis. The first of those enzymes is pyruvate carboxylase, which is allosterically activated by acetyl CoA derived from fatty acid oxidation. The fatty acid oxidation rates are elevated in T2D because it is required for ATP production due to the lack of glucose uptake. The second and fourth gluconeogenesis regulatory enzymes, phosphoenolpyruvate carboxykinase (PEPCK) and glucose-6-phosphatase, are regulated by insulin at the transcriptional level [86]. Finally, the third regulatory enzyme, fructose-1,6-bisphosphatase, is activated by citrate, which is elevated as a consequence of higher fatty-acid-derived acetyl-CoA levels, as the initial product of the citric acid cycle. Thus, insulin resistance leads to the dysregulation of hepatic gluconeogenesis in people with diabetes, even in post-prandial conditions, thus exacerbating chronic fasting hyperglycemia [4,87].

### 2.9. Estrogen

Though it is typically regarded as a sex hormone, estrogen has been shown to play a role in the regulation of glucose uptake and insulin signaling [88]. Estrogen can stimulate GLUT4 translocation, lower inflammation, decrease the oxidation of lipoproteins, and decrease the number of triacylglycerols in the bloodstream [88]. It also stimulates cAMP, which regulates several metabolic mediators, including the release of calcium [88]. 

T2D has exhibited a trend of being more prevalent in males than females [87], and the fact that estrogen exists in much higher levels in females may partially explain this phenomenon. Though the issue is far more complex than estrogen alone, the key role of estrogen in maintaining glucose homeostasis is partly evidenced by an increased incidence of T2D in post-menopausal women compared to pre-menopausal women [87]. Menopause is a time of drastic transition and many hormonal changes in women [89]. One of the major results of menopause is a marked decrease in estrogen levels [90]. The post-menopausal levels of triacylglycerols are much higher than the triacylglycerol levels in pre-menopausal woman [89]. Menopause has also been shown to contribute to obesity and increased pro-inflammatory cytokine production, such as TNF-α [89]. A few of the polyphenols (lignans) found in coffee are phytoestrogens, meaning they mimic some of the effects of estrogen and are therefore suitable topics for research as potential anti-T2D agents [91].

## 3. Coffee-Derived Polyphenols as Intercessors of T2D Pathogenesis

### 3.1. Polyphenols

Polyphenols are naturally occurring secondary plant metabolites found in every part of most plants. Coffee contains over a thousand compounds, with an average of 355 mg of polyphenols in a 180 mL serving [15]. This number can be anywhere from 1.5 to 5 times higher in espresso [15]. The extent to which polyphenols appear in the beverage depends on several factors, including the roasting process [92] and method of preparation [93]. Polyphenols have been shown to have a large variety of health benefits [94]. They have been shown to decrease biomarkers of inflammation [95], exert positive hormonal effects on menopausal women [91], and are associated with improved breast cancer prognosis [96,97]. The four main sub-classes of polyphenols are flavonoids, lignans, phenolic acids, and stilbenes, all of which can be found in a variety of common food sources, including tea, coffee, whole grains, fruits, wine, flaxseed, and berries [98,99]. In fact, coffee has been shown to contain the highest concentration of polyphenols per serving of any beverage [100]. Most of the compounds discussed in this review fall under the category of lignans. Lignans are secondary metabolites that are formed when phenylpropanoids dimerize through oxidation [101]. They are natural antioxidants [102] and have demonstrated potential to moderate diabetic hyperglycemia [103], reduce bodyweight [104], and lower blood pressure [103].

### 3.2. Lignans and Their Metabolites

#### 3.2.1. Secoisolariciresinol

Secoisolariciresinol (SL) is a plant lignan found in various food sources and is especially abundant in flaxseed [105,106]. Because it is one of the few lignans with an oligomeric structure, it is classified as a lignan macromolecule [107]. SL was shown to prevent the onset of diabetes in streptozotocin (STZ)-induced diabetic mice in 75% of a testing sample [73]. There are several proposed mechanisms through which SL could have elicited this effect. First, SL has been shown to convey antioxidant properties, which is a common characteristic among polyphenols. More specifically, it has been posited to work as an ROS scavenger, thereby decreasing the production of pancreatic malondialdehyde, which is an indicator of ROS-mediated cytotoxicity [73]. SL has also been shown to play a role in the activation of nuclear Nrf2, which increases the expression of hemeoxygenase-1 and Nqo1, both of which have antioxidant properties [108,109]. SL-mediated diabetes prevention has also been associated with the preservation of the reserve of pancreatic antioxidative agents in diabetic rats [73]. Thus, SL may convey a protective effect on pancreatic beta-cells and perhaps attenuate the oxidative stress and resultant inflammation that is associated with T2D pathogenesis [73].

SL has also been shown to regulate the interactions between AMPK and FOXO3 [110] (Table 1 and Table 2). A decreased expression of FOXO3a has been shown to decrease apoptotic activity, while the increased phosphorylation, and thus activation, of AMPK (Thr172) leads to an increased glucose uptake and lower levels of biomarkers of inflammation [110] (Table 1). SL was shown to decrease the FOXO3a and Bax levels while increasing the Bcl-2 protein levels in cardiomyocytes [111] (Table 2, Figure 2). This study was conducted in vivo in 30 male rats at a concentration of 20 mg/kg bodyweight and in vitro with 500 µM of secoisolariciresinol diglucoside (SDG) in H9c2 cardiomyocytes [110,111]. These changes in protein levels correlate with lower incidence rates of apoptosis and inflammation [111]. 

Additionally, SL has been shown to increase the phosphorylation of Akt and IRS-1 in hepatocytes and C57BL/6J male mice with varying SDG concentrations (10, 100, and 1000 mg/kg/d) [112,113], which may contribute to improved glucose homeostasis by inhibiting glycogenolysis and gluconeogenesis. Indeed, SL has been suggested to lower glucose levels by decreasing PEPCK expression [114,115], which is a rate-limiting enzyme in gluconeogenesis [115] (Table 1). The PEPCK study was performed in a primary hepatocyte culture using 100 µM of SDG [115]. The activation of IRS-1 and Akt is also known to promote GLUT4 translocation in adipocytes and skeletal muscle. Because SL has been shown to contribute to the positive regulation of AMPK, Akt, and IRS-1 and the inhibition of FOXO3a, it is likely that it either directly phosphorylates IRS-1 or a protein in the AMPK/GLUT4 pathway (Figure 1). However, further research is needed to more clearly discern this mechanism. These findings collectively suggest that SL has the potential to promote euglycemia both indirectly by inhibiting the exacerbation of hyperglycemia caused by hepatic insulin resistance and directly by promoting glucose disposal [73,115].

#### 3.2.2. Enterodiol

Enterodiol (EDL) is a mammalian gut metabolite of SL, which involves a series of steps facilitated by the gut microflora [116,117]. The deglycosylation of SL-diglucoside (SDG) by β-glycosidases, produced by bacteria such as *Bacteroides distasoni*, forms SL [118]. Subsequent demethylation and dihydroxylation steps facilitated by bacteria, such as *Butyribacterium methylotrophicum* and *Clostridium scindens*, respectively, results in the final product of EDL [118]. EDL is also the oxidation product of another phytoestrogen, enterolactone (ENL) (subsequently discussed) [119]. Both EDL and ENL have phenolic hydroxy groups located only at the *meta*-position, distinguishing them from their plant lignan counterparts [91]. 

As it pertains to T2D, EDL has been shown to inhibit triacylglycerol uptake in HEPA1-6 cells and adipogenesis in fat cells [5] (Table 3). In fact, a recent study using colorectal cancer cells indicated the association of EDL with the decreased phosphorylation of ERK, JNK, and the p38 subunit of MAPK, all of which inhibit the MAPK pathway [116] (Table 1, Figure 1). These effects were shown to slow the progression of colorectal cancer [116]. While this study most directly pertains to cancer, these effects are very much related to the progression of T2D as well. The downregulation of the MAPK pathway has been correlated with the increased translocation of GLUT4 to the cell membranes of adipocytes and skeletal muscle cells, thus leading to increased glucose uptake and attenuating hyperglycemia [65] (Table 1).

Additionally, like ENL, the presence of EDL has also been shown to inhibit NF-κB, [120]. Another condition associated with T2D is leaky gut syndrome, in which lipopolysaccharide (LPS) passes from the intestinal lumen, between the enterocytes, into the blood stream and causes a state of chronic low-grade inflammation. LPS accomplishes this by causing the degradation of I-κB, an inhibitor of NF-κB [120]. Both EDL and ENL have been shown to prevent I-κB degradation, thus indirectly inhibiting the effects of NF-κB and contributing to lower inflammation [120] (Table 2; Figure 2). 

#### 3.2.3. Matairesinol

Matairesinol (ML) is a plant-derived dibenzylbutyrolactone lignan and, as previously mentioned, is the precursor for ENL and an indirect precursor for EDL [91,121]. ML has been shown to slow the progression of bodyweight gain, increased fat mass, adipocyte size, and hepatic fat deposition, which are commonly associated with T2D [5]. Though its effects on glucose disposal have not yet been directly reported, its gut metabolite, ENL, has been shown to be effective [122], thus suggesting that ML might also have the potential to promote blood glucose homeostasis. Indeed, ML administration caused a 70% decrease in insulin secretion and improved glucose tolerance as well as insulin sensitivity in a high-fat-diet-fed adipogenesis mouse model [5]. This was performed in vitro using 3T3-L1 fibroblasts with concentrations of 0.01, 0.1, and 1 µM [5]. In addition, biomarkers of inflammation including IL-6 and TNF-α expression were attenuated by ML administration [5] (Table 2). The mechanism by which ML promotes these effects is not completely clear, as the study noted elevated serum levels of EDL and ENL [5], suggesting that the metabolites of ML are most directly responsible for these biological effects. Further, it was shown that compared to ML, EDL and ENL had greater effects on lipid accumulation inhibition in adipocytes and hepatocytes in vitro [5] (Table 2). Despite the effects on lipid metabolism being most likely facilitated by the metabolites of ML, it is still worth conducting a future investigation to further elucidate the means by which ML promotes glucose tolerance, perhaps by evaluating its effect on acute insulin secretion and glucose disposal. 

#### 3.2.4. Enterolactone

ENL is the oxidation product of ML and, like EDL, is formed in the mammalian gut from its plant lignan precursor [91]. ENL has been shown to promote glucose uptake in skeletal muscle [122]. This was demonstrated in an in vivo study with male db/db mice using 0.001% and 0.01% ENL as well as an in vitro study of L6 myocytes using concentrations of 0, 25, 50, and 100 µM ENL [122]. It has also been shown to inhibit hepatic triacylglycerol uptake and adipogenesis in adipocytes [123] (Table 3). Moreover, there is a negative correlation between T2D and the presence of ENL in urine, indicating ENL’s potential anti-diabetic effects [122]. The mechanisms by which ENL accomplishes this have been studied in the skeletal muscle of mice [122]. ENL does not increase glucose uptake when an AMPK inhibitor is introduced [122]. While the specific mechanisms from which ENL elicits its affects are still being characterized, the marked decrease in glucose disposal when an AMPK inhibitor is introduced advances the notion that ENL is somehow responsible for the phosphorylation and activation of AMPK and utilizes a GLUT4-dependent mechanism for glucose uptake (Table 1). 

ENL, similarly to other lignans and metabolites, has demonstrated abilities to modulate the activity of cellular signaling pathways involved in cancer cell proliferation and cell cycle progression. Primarily, ENL’s regulation of CREB, ERK1/2, Bcl-2, and p38 signaling have been reported as the most evaluated mechanisms in which ENL attenuates pathological states [124,125,126] (Table 1 and Table 2). Studies evaluating the efficacy of ENL have found significant decreases in cancer cell metastasis, proliferation, angiogenesis, and invasion through the direct inhibition of the MAPK-p38 pathway [127,128,129]. This has been demonstrated in MDA-MB-231 breast cancer cells using 25, 50, and 75 µM concentrations of ENL [127]. CREB was also significantly inhibited by ENL, leading to a decrease in the transcription of genes associated with inflammatory signaling [130] (Figure 2). The direct modulation of the PI3K/Akt and insulin-like growth factor-1 receptor (IGF-1R) pathways have also been examined, both of which play key roles in cancer cell proliferation [131,132,133]. This has been demonstrated in PC-3 prostate cancer cells using concentrations between 20 and 60 µM ENL [133]. 

The ability of ENL to directly bind and inhibit key proteins and receptors that are necessary for cancer development, when coupled with its reported regulation of cytokine production, demonstrates its potential to attenuate pathologies such as T2D. Pathologies such as T2D require the production of pro-inflammatory cytokines, such as TNF-α, IL-1, 6, 8, and IKKβ [134]. ENL has shown to directly inhibit TNF-α, NF-κB, and IKKβ while indirectly inhibiting IL-1, 6, and 8 in addition to other pro-inflammatory cytokines [120] (Table 2). This was determined using THP-1 cells and varying concentrations between 0 and 1 mM [120].

These findings have propelled the applications of ENL into more clinical settings. In addition to estrogen-dependent cancer treatments, ENL has been looked at as a potential mediator for post-menopausal symptom onset [135]. Estrogen regulates the production of luteinizing hormone (LH) and follicle-stimulating hormone (FSH) through a negative feedback inhibition [135]. Once menopause occurs, estrogen secretion decreases, leading to LH and FSH dysregulation and the onset of inflammatory symptoms such as skin flushing, hypercholesterolemia, and atherosclerosis [135]. ENL, a phytoestrogen, has been reported to bind to estrogen receptors, such as ERα and Erβ, to reduce cardiovascular pathologies [135]. Estrogen-mediated receptors such as these have been shown to exhibit an affinity for ENL and other lignans. Studies have also shown that these estrogen receptors, being more prevalent in patients with T2D, also modulate the translocation and expression of GLUT4 proteins [136]. Akin to the previously mentioned AMPK/GLUT4 hypothesis, there also exists the proposal that estrogen-mediated macronutrient disposal may serve as a target for future research with ENL and lignan efficacy driving the molecular mechanisms. Each of these future research aims, when coupled, can compound the regulation of insulin-sensitive tissues and demonstrate a multifaceted approach to elucidating glucose homeostasis in patients with T2D. 

### 3.3. Flavonoids

#### 3.3.1. Kaempferol

Kaempferol (KL) is another polyphenol found naturally in coffee and has been shown to have several health benefits, including being anti-inflammatory [137] and anti-hypertensive [60]. Studies confirming its anti-hypertensive properties were performed both in vitro in primary human skeletal muscle cells with 10 µM of KL and in vivo using skeletal muscle tissue collected from C57BL/6 male mice treated with 50 mg/kg of bodyweight [60]. In the context of T2D, KL has been shown to regulate the lipid metabolism [138,139,140,141,142], promote blood glucose homeostasis [143,144,145,146,147], improve the functional β-cell mass [76,142,148,149,150,151], and promote a gut flora profile that has been shown to be consistent with [152]. Studies demonstrating the KL-mediated regulation of the lipid metabolism were performed in vitro on 3T3-L1 adipocytes with 60 µM of KL [141] and in vivo in male C57BL/6J mice with 10 mg/mL bodyweight [140]. Its promotion of blood glucose homeostasis was shown in vivo with apolipoprotein-E-deficient mice treated with 150 mg/kg of bodyweight [143], male Wistar rats treated with 50, 100, and 200 mg/kg bodyweight (kaempferitrin) [144], and with C57BL/6 mice treated with 50 mg/kg of bodyweight [147]. The ability of KL to improve the β-cell mass was shown in INS-1E cells treated with 0.1, 1, and 10 µM of KL [76,150,151] and on RIN-5F cells treated with 10 µM of KL [148]. Its promotion of a healthy gut flora profile was demonstrated in C57BL/6 cells treated with 200 mg/kg of bodyweight [152]. 

To elicit such a broad range of anti-diabetic activity, it is likely that KL either employs a multi-faceted range of mechanistic activity or regulates a central regulator of multiple metabolic pathways. Indeed, multiple studies have shown that KL works by activating AMPK [60,148,149]. This was demonstrated in primary human skeletal muscle cells treated with 10 µM of KL and in C57BL/6 male mice treated with 50 mg/kg of bodyweight [60]. AMPK is activated allosterically by an increased ratio of AMP:ATP as well as covalently by several mediators, including LKB1 and calmodulin-dependent kinase kinase β (CAMKKβ), which is activated by increased levels of calcium. Thus, AMPK is activated by various indicators of cellular stress and a negative energy balance. 

As it pertains to the regulation of fat metabolism, AMPK activates malonyl-CoA decarboxylase (MCD) and inhibits acetyl-CoA carboxylase (ACC). In the post-prandial state, ACC converts acetyl-CoA to malonyl-CoA, which blocks carnitine palmityl transferase 1 (CPT1), thus preventing the entry of fatty acids into the mitochondria, where they would otherwise be oxidized. In the fasted state, MCD converts malonyl-CoA back to acetyl-CoA, which then allows CPT1 to facilitate the transport of fatty acids across the mitochondrial membrane complex to allow for β-oxidation. Thus, by activating AMPK, KL has been shown to covalently inhibit ACC in adipose, liver, and muscle tissues, thereby promoting fatty acid oxidation [153]. In addition, KL has been shown to stimulate lipolysis by promoting the expression of adipose triacylglycerol lipase and hormone-sensitive lipase [141] (Table 3). This was shown in 3T3-L1 adipocytes treated with 60 µM of KL [141].

In addition to promoting the catabolism of stored fat, kaempferol has also been shown to stimulate glucose uptake in skeletal muscle via an AMPK-dependent mechanism, perhaps via the PI3K/Akt pathway, in a manner that is independent of the insulin receptor [60] (Table 1). This is consistent with the findings from an animal study, in which STZ-induced diabetic rats that were administered with KL had reduced blood glucose levels [154]. This study also showed that the plasma insulin levels were higher in rats treated with KL [154]. As AMPK is activated by CAMKKβ, which is activated by elevated Ca^2+^ levels, these data collectively support the rationale that KL may act to regulate intracellular calcium levels. Indeed, KL has been shown to activate a mitochondrial calcium uniporter [155], which plays a role in directing glucose-stimulated insulin secretion in pancreatic β-cells [150]. KL has also been shown to activate PDX-1 in INS-1E cells treated with 0.1, 1, and 10 µM of KL, which was consistent with increased cAMP, which is also required for insulin secretion [76] (Table 2, Figure 2). PDX-1 is also regulated by AMPK [156], further suggesting its role in KL-mediated glucose homeostasis.

Despite the wide array of data surrounding the mode of action of KL, whether it promotes AMPK activity directly or indirectly by activating a complex upstream, such as CAMKKβ, remains unknown [60] and warrants further investigation. A recent study also showed that KL may potentially decrease T2D risk and increase glucose uptake by activating PDX-1, which, as described above, can improve β-cell health and promote glucose uptake into the cell from the bloodstream [36].

#### 3.3.2. Quercetin

Quercetin (QN) is another polyphenol found in coffee, with health benefits ranging from neurological to metabolic [157]. A recent study, performed on both human THP-1 monocytes and U373 astrocytoma cells treated with 0.1, 0.3, 1, 3, 10, 30, 100, 300, and 1000 µg/mL concentrations of KL, showed that 100 g of coffee beans included approximately 200 mg of QN [157]. One study showed that QN diminished the TNF-α-induced expression of pro-inflammatory mediators COX-2, iNOS, NF-kB, p65, and VCAM-1 [158] (Table 2). QN has also been demonstrated to effectively attenuate inflammation and activate cellular pathways that promote the neutralization of ROS, including the p38 MAPK pathway in skeletal muscle [159]. This study was performed on L6 myoblasts treated with 10 and 100 µM of KL [157]. QN has also been shown to inhibit the release of TNF-α and IL-6, resulting in the inhibition of the p38 MAPK pathway [145,157] (Table 1, Figure 2). QN has also been shown to elicit antioxidant effects by increasing glutathione production in glial cells, which serves as a reducing agent in the neutralization of ROS [157]. The ability of QN to neutralize ROS and decrease the secretion of pro-inflammatory mediators may have anti-diabetic implications and should be further investigated in this context. Indeed, several polyphenols have been shown to attenuate inflammation-mediated insulin signaling dysregulation [160]. More specifically, resveratrol, like QN, has been shown to increase diabetes-mediated decreases in glutathione levels, which has been linked to improvements in diabetic retinopathy [161]. Because of the structural and mechanistic similarities, QN may also play a role in attenuating secondary T2D sequelae, such as diabetic retinopathy.

### 3.4. Phenolic Acids

#### Chlorogenic Acids

The structure of chlorogenic acid (CA) encompasses both caffeic and quinic acids, as it is an ester of the two [162]. CA is formed in plants throughout the process of aerobic respiration and has been utilized in traditional Chinese medicine for some time [163]. CA is the most abundant of all the polyphenols found in coffee [164], comprising 5–10% of the coffee bean, which is substantial given that caffeine only comprises 1–2% [165]. CA has been shown to have multiple health benefits, including decreased levels of plasma triglycerides and cholesterol, the slowing of weight gain, reduced hyperglycemia, and attenuated insulin resistance [163]. 

Exposing HepG2 cells to CA resulted in greater insulin sensitivity [163]. In skeletal muscle cells, CA attenuated an insulin-induced blunting of IRS-1 expression, as well as PI3K/Akt activation, which translated to increased GLUT4 levels [163] (Table 1, Figure 1). As seen with KL studies, the effects of CA on these mediators of glucose homeostasis were likely dependent on AMPK, as knocking out AMPK significantly blunted the CA-mediated results [163,166]. This effect was seen in both in vivo and in vitro studies treating male db/db mice with 250 mg/mL of CA and L6 mytotubes with 2 mmol/L of CA, respectively [166]. This suggests that the role of CA in stimulating GLUT4 translocation to the membrane is the phosphorylation and activation of AMPK [163] (Table 1). Another study performed by treating male Sprague Dawley rat muscle with 1 mM of CA further indicated that caffeic acid, a metabolite of CA, promotes AMPK phosphorylation in skeletal muscle [167]. While these data are intriguing, more work needs to be conducted to distinguish between the effects elicited by caffeic acid and those elicited by CA, as CA is readily converted into caffeic and quinic acids [167]. Among the chlorogenic acids are a variety of other acids, including caffeoylquinic acid (CQA), feruloylquinic acid, and *p*-coumaaroylquinic acid, which possess antibacterial, anti-inflammatory, and antioxidant properties [168]. More specifically, CQA has demonstrated many anti-diabetic effects, including decreased bodyweight, lower blood glucose levels, and improved inflammation [169]. This study was performed on 60 db/db mice and 12 C57BL/6 mice by treating them with 50, 100, and 200 mg of *Pandanus tectoruis* fruit containing large quantities of CGA per kg bodyweight [169]. This was also accomplished through the phosphorylation of AMPK and Akt, increasing the presence of GLUT4 transporters in the skeletal muscle [169]. Additionally, it upregulated the activity of hexokinase, a major player in the glycolysis pathway, while decreasing that of glucose-6-phosphate and phosphoenolpyruvate carboxykinase, hindering the process of gluconeogenesis [169] (Table 1, Figure 1). This further contributes to lower glucose levels in the blood and slows the progression of T2D. 

### 3.5. Stilbenes

Stilbenes have also been found to exist naturally in coffee. Specifically, resveratrol (RTL) and dihydroxy-resveratrol (DR2) have been extensively investigated as potential anti-diabetic agents [170,171].

Clinically, RTL has been shown to significantly reduce HbA1c, inflammation, and oxidative stress while also restoring homeostatic blood glucose regulation in patients with T2D [172]. Specifically, significant reductions in IL-6, TNF-α, and highly sensitive-C reactive protein were reported after a 24-week protocol at 200 mg of RTL/day in patients with diabetes [172]. With respect to glucose regulation, similar to the lignans, RTL has demonstrated an ability to promote the expressions of AMPK, Akt, PI3K, and GLUT4 in rat models [173,174,175]. These studies were performed on male Sprague Dawley rats treated with 2.5 mg/kg of RTL [173] and male Wistar rats treated with 0.05, 0.1, 0.5, 3.0, 6.0, and 10.0 mg/kg of RTL [175].

DR2, a gut-microbial metabolite of RTL, has also been studied for its potential in the attenuation of metabolic and endocrine pathologies. Recent findings show that DR2 activates AMPK/SIRT1 signaling proteins, which effectively inhibit p38/MAPK proteins, blunting adipocyte maturation in 3T3-L1 adipocytes [176]. Moreover, DR2 exhibits efficacy in modulating intracellular lipid accumulation through the phosphorylation of ACC, alleviating lipid peroxidation in hepatocytes, and enhancing insulin sensitivity via an Akt-dependent manner in insulin-resistant HepG2 and C2C12 cell lines [176]. This study was performed on 3T3-L1 adipocytes treated with 20, 40, and 80 µM of DR2 or 40 µM of RTL and on human hepatocarcinoma HepG2 cells treated with 10, 20, and 40 µM of DR2 [176]. DR2 has also been shown to reduce the expression of secreted embryonic alkaline phosphatase proteins by activating the TLR-4/NF-κB signaling pathway [177]. This study was performed on male CD-1 mice treated with 4.6 mg/kg of RTL [177]. Overall, the effects of stilbenes, with RTL and DR2 being those most evidenced to affect T2D pathogenesis, have been well reviewed [178,179,180,181,182,183] and are viable candidates for further investigation as to their potential to ameliorate T2D.

### 3.6. Factors Affecting Polyphenol Content in Brewed Coffee

#### 3.6.1. Roasting

The majority of the coffee that can be purchased in a typical market is produced from two species, *Coffea arabica* (Arabica) and *Coffea canephora* syn *Coffea robusta* (Robusta). As a rule, the antioxidant capacity and polyphenol content are higher in Robusta coffee compared to Arabica coffee [184]. However, other studies have shown that the roast level (light, medium, or dark) is a greater determinant of the polyphenol content in the final consumed beverage than the type of coffee [185]. During the first few minutes of roasting, phenolic compounds increase because of the high temperature promoting the generation and release of antioxidants [186], but subsequently decrease with increased roast levels [184]. Thus, lighter roasted coffee has the largest percentage of polyphenols and antioxidants present [164]. Of course, green coffee contains a higher concentration of polyphenols compared to medium and darker roasted coffee, but roasting is widely considered to improve the sensory quality [187].

#### 3.6.2. Method of Brewing

The method of brewing affects the polyphenol composition of coffee, though some studies show that the difference is more drastic than others. Several popular methods of brewing include the use of a drip maker, French press, pour over (V60), percolator, and AeroPress [188]. Interestingly, despite Robusta beans being higher in polyphenols than Arabica beans, changing the brewing method can allow for the extraction of a similar or even higher percentage of polyphenols from Arabica beans [189]. Specifically, a drip maker can allow for a very similar concentration of polyphenols, while using a percolator may allow for an even higher percent extraction of polyphenols from Arabica beans [188,189]. One study comparing the effects of a drip maker, percolator, and AeroPress showed that the AeroPress gave significantly lower yields of total phenolic compounds and lower antioxidant activity compared to the other two methods [188]. Another study showed that the percolator, compared to a drip maker and pour over method, allows for the greatest extraction of polyphenols regardless of whether the beans are Arabica, Robusta, or even decaffeinated [189]. The percolator has also been shown to be more effective in producing a brew that is higher in antioxidant capacity compared to the drip maker and the French press; however, the same study found that the percolator did not yield a significantly higher concentration of phenolic compounds than either the French press or the drip maker [190]. Studies have also shown that phenolic compounds are isolated to a greater extent with the espresso method compared to other methods, including lungo and ristretto, which are subcategories of espresso [190,191]. 

#### 3.6.3. Organic Cultivation and Common Processing Methods

Interestingly, the presence of bioactive compounds in general, including phenolic compounds and polyphenols, especially of CA, was found to be greater in organic coffee than in normal coffee [164]. It is hypothesized that the lack of mineral fertilizers and pesticides causes the plants to synthesize more polyphenols in order to protect themselves and thrive, as polyphenols are known to be natural pesticides for the plant [164,192,193]. 

The so-called coffee “bean” is a processed seed from a cherry-like fruit. The three most common methods of processing the fruit to the end of harvesting the seed, which is subsequently roasted, are honeying, natural, and washing. The honeying process removes the skin and part of the pulp of the ripe fruit and then leaves the bean, still containing some of the pulp, to dry for roughly two weeks on African raised beds. The natural process allows the entire intact coffee cherry to be left to dry on African raised beds in the sun, which takes roughly five weeks. The washing method completely de-pulps the fruit and then lets the bean soak and ferment for roughly two days before washing and cleaning any remaining fruit and leaving the bean to dry. While the research is limited as to how this affects the polyphenol content, one recent study showed that the highest polyphenol content of these three processes was found in washed Nicaraguan beans, with the lowest polyphenol content being found in honeyed Nicaraguan beans [194]. Interestingly, the CA content was the highest in naturally processed Peruvian beans, with the honeyed Nicaraguan beans containing the smallest CA content [194]. The CA content in the beans with these processing methods was consistent with the overall antioxidant capacity [194]. The limitations of this study are that not all of the analyzed beans were processed in all three ways. Thus, the differences could be a result of the origin of the coffee beans rather than the processing method. Further research should be conducted to more definitively determine which of these processing methods yields the highest polyphenol content and oxidative capacity. 

#### 3.6.4. Milk

Coffee is often consumed with milk to mitigate the perceived bitter taste. Many typical espresso-containing beverages, including lattes, flat whites, and macchiatos, are mostly milk. This begs the following question: does the addition of milk affect the beneficial properties of coffee? While the available research is limited, the contents of total phenols and CA as well as the antioxidant capacity have indeed been shown to significantly decrease in coffee with the addition of milk [195]. This is thought to be due to the chemical nature of casein, which is the major class of proteins found in dairy products, being hydrophobic with a high charge [196]. It has been suggested that non-covalent interactions between proteins and phenolic compounds are largely hydrophobic interactions, which are stabilized by hydrogen bonds [197]. A more recent study using green tea flavonoids demonstrated that this is indeed the case [198]. Thus, more work is needed in this field to specifically determine the extent to which the biological effects of each individual compound are affected by milk. However, the currently available data suggest that black coffee is the most conducive to reaping the maximal potential health benefits from the polyphenols and antioxidants found in coffee. 

## 4. Conclusions and Future Directions

Overall, each of these compounds has been shown to convey some sort of anti-diabetic effect, whether it is directly related to glucose uptake and homeostasis, inflammation, β-cell protection, or some other mechanism. The available research suggests that the polyphenols found in coffee convey strong anti-inflammatory and antioxidant effects. Specifically, EDL and QN have been shown to inhibit the p38 subunit of the MAPK pathway, thus downregulating the production of inflammatory cytokines such as TNF-α and IL-6. SL has the ability to attenuate the production of ROS and subsequent β-cell damage. Most of the polyphenols discussed are further able to promote β-cell health in some way. KL has the ability to stimulate the activation of PDX-1, eventually improving the functional β-cell mass and function. EDL increases the expression of GLUT2, which can lead to decreased blood glucose by promoting an increased intracellular ATP:ADP ratio that culminates in cAMP activation and insulin secretion. ENL, CL, and CA have all been demonstrated to affect the AMPK pathway. This activation of AMPK allows PIP2 to be phosphorylated to PIP3, triggering the PI3K/Akt pathway, which promotes the translocation of GLUT4 to the cell membrane, subsequently facilitating glucose disposal in adipocytes and skeletal muscle tissue.

Because many of these compounds have been studied in relation to their anti-diabetic effects on skeletal muscle, it would be beneficial to elucidate these effects and their mechanisms in adipose tissue as well. Adipose tissue is largely responsible for glucose uptake and plays a significant endocrine role with respect to metabolic regulation [199]. For example, leptin and adiponectin are both produced by adipose tissue and have the potential to exhibit anti-diabetic effects [35]. A recent study indicated increased adiponectin and decreased leptin levels in response to coffee consumption [35]. The same study showed an anti-inflammatory effect by promoting increased levels of anti-inflammatory cytokines and decreased levels of pro-inflammatory cytokines [35]. The potential that the mechanisms of the reviewed polyphenols contributed to this effect is very likely. Because compounds such as EDL and ENL are phytoestrogens, the hormonal effects could compound with the metabolic effects in both positive and negative ways. Especially because the loss of estrogen in postmenopausal women results in an increase in T2D, the mechanisms through which these compounds work could be in a manner similar to estrogen [87]. Knowing and understanding how these compounds influence various systems and types of tissue is the next step in pursuing this as a potential means of T2D treatment and can even elucidate new benefits. Additionally, other than the fact that these compounds naturally exist together in various food items, their synergistic effects have not been extensively tested. This is another warranted area of future work that is necessary to maximize the efficacy of the use of polyphenols in mitigating T2D.

To apply these data in terms of coffee consumption, as with most naturally occurring compounds found in food sources, it is unlikely that the levels of these compounds used in these studies can be achieved merely by consuming more coffee. That is not to say that there may be some benefit to coffee consumption. While there are many variables involved in bringing a coffee bean from a farm to a cup, studies aiming to investigate this are limited. However, the available data seem to suggest that espresso or a percolator for non-espresso brews, brewing an organic, lightly roasted coffee, which was processed either by the washing or natural method, without milk, may be the optimal method in terms of achieving the highest levels of polyphenols and antioxidant capacity. Research seems to suggest that the addition of milk may be the greatest hindrance to achieving the maximum health benefits of coffee. Other than that, the summation of the available literature suggests that cultivation/processing/roasting/brewing methods do not significantly affect the potential health benefits of coffee. Thus, coffee is an excellent source of polyphenols and antioxidants regardless of most of these variables. Other variables that may affect the polyphenol content and antioxidant capacity of a coffee beverage, including the origin of the beans [188], are not reviewed here due to either lacking significant associations with the polyphenol content or a scarcity of works in the literature regarding their effects.

## Figures and Tables

**Table 1:** Summary of mechanisms through which each compound directly affects blood glucose levels are highlighted. (+) indicates a positive effect and (−) indicates no significant effect. (*) denotes an in vitro study, while (#) denotes an in vivo study.

	PI3K/Akt/GLUT4 (Activation)	AMPK (Activation)	MAPK (Inhibition)	PEPCK (Inhibition)	ERK (Activation)
EDL	+ ^#^	−	+ *	−	+ *
ENL	+ *	+ *^#^	+ *	−	+ *
ML	−	−	−	−	−
SL	+ *^#^	+ ^#^	−	+ *	−
KL	+ *^#^	+ *^#^	−	−	−
QN	−	−	+ *^#^	−	−
CA	+ ^#^	+ *^#^	−	+ ^#^	−

**Table 2:** Summary of mechanisms through which each compound attenuates T2D-associated pathways in the context of inflammation and pre-mature β-cell death. (+) indicates a positive effect, and (−) indicates no significant effect. (*) denotes an in vitro study, while (#) denotes an in vivo study.

	NF-κB (Inhibition)	TNF-α (Inhibition)	Bcl-2 (Activation)	FOXO-3a (Inhibition)	PDX-1 (Activation)
EDL	+ ^#^	−	−	−	−
ENL	+ *	+ *	+ *	−	−
ML	−	+ *	−	−	−
SL	−	−	+ *	+ *^#^	−
KL	−	−	−	−	+ *
QN	+ *	+ *	−	−	−
CA	−	−	−	−	−

**Table 3:** Summary of mechanisms through which each compound contributes to the attenuation of select common T2D sequelae. (+) indicates a positive effect and (−) indicates no significant effect. (*) denotes an in vitro study, while (#) denotes an in vivo study.

	Triacylglycerol Uptake (Inhibition)	Lipid Accumulation (Inhibition)	Adipose Triacylglycerol Lipase (Activation)	Hormone Sensitive Lipase (Activation)
EDL	+ *	+ *	−	−
ENL	+ ^#^	+ ^#^	−	−
ML	−	+ *	−	−
SL	−	−	−	−
KL	−	−	+ *^#^	+ *
QN	−	−	−	−
CA	−	−	−	−
